# Modeling hind-limb kinematics using a bio-inspired algorithm with a local search

**DOI:** 10.1186/s12938-018-0565-6

**Published:** 2018-11-20

**Authors:** S. Ivvan Valdez, Josué González-Sandoval, Sergio Dueñas-Jiménez, Nancy Elizabeth Franco Rodríguez, Sulema Torres-Ramos, Gerardo Mendizabal-Ruiz

**Affiliations:** 1 División de Ingenierías, Universidad de Guanajuato, Carr. Salamanca-Valle de Santiago km 3.5+1.8, 36885 Salamanca, Guanajuato México; 2 Departamento de Ciencias Computacionales, Universidad de Guadalajara, Av.Revolución 1500, Guadalajara, Jalisco México; 3 Departamento de Neurociencias, Universidad de Guadalajara, Sierra Mojada 950, Guadalajara, Jalisco México; 4 Departamento de Farmacobiología, Universidad de Guadalajara, Blvd. Marcelino García Barragan, 1421 Guadalajara, Jalisco México

**Keywords:** Genetic algorithm, Local search, Hind limb, Laboratory rats, Kinematics analysis

## Abstract

**Background:**

Laboratory rats play a critical role in research because they provide a biological model that can be used for evaluating the affectation of diseases and injuries, and for the evaluation of the effectiveness of new drugs and treatments. The analysis of locomotion in laboratory rats facilitates the understanding of motor defects in many diseases, as well as the damage and recovery after peripheral and central nervous system injuries. However, locomotion analysis of rats remains a great challenge due to the necessity of labor intensive manual annotations of video data required to obtain quantitative measurements of the kinematics of the rodent extremities. In this work, we present a method that is based on the use of a bio-inspired algorithm that fits a kinematic model of the hind limbs of rats to binary images corresponding to the segmented marker of images corresponding to the rat’s gait. The bio-inspired algorithm combines a genetic algorithm for a group of the optimization variables with a local search for a second group of the optimization variables.

**Results:**

Our results indicate the feasibility of employing the proposed approach for the automatic annotation and analysis of the locomotion patterns of the posterior extremities of laboratory rats.

**Conclusions:**

The adjustment of the hind limb kinematic model to markers of the video frames corresponding to rat’s gait sequences could then be used to analyze the motion patterns during the steps, which, in turn, can be useful for performing quantitative evaluations of the effect of lesions and treatments on rats models.

## Background

Laboratory rats play a critical role in research because they provide a biological model that can be used for evaluating the affectation of diseases and injuries, and for the evaluation of the effectiveness of new drugs and treatments [1–3]. In particular, the analysis of locomotion in laboratory rats allows us to understand the motor defects in diseases such as osteoarthrosis [4], Parkinson’s disease, Huntington’s disease, and amyotrophic lateral sclerosis [5, 6], as well as the damage and recovery after peripheral and central nervous system injury [7].

Current methodologies for the analyses of laboratory rats’ locomotion can be categorized as “forced” when the speed of march is imposed on the rat by means of a treadmill (e.g., [8]), or “unforced” when the rat is free to move at any speed on the ground or an activity wheel [7]. In general, forced analysis have the advantage of allowing to perform direct comparisons between the recollected gait variables, since all of the subjects will be moving at similar speeds. However, for some research cases, these strategies may be unsuitable, since the movement of the rats could be different from their normal locomotion patterns [9]. For these cases, unforced methods may be a better option with the main disadvantage that the comparison of the gait variables would require a more elaborated metric, exclusion of samples, or a normalization of the data.

There currently are qualitative methods for the assessment of locomotion variables, such as the Tarlov scale [10] and the inclined plane test [11], and the Basso, Beattie and Bresnahan (BBB) rating [12]. BBB is the most widely accepted method for assessing locomotion on unforced-open field tests. The BBB rating consists of a semi-quantitative scale which takes values ranging from zero to twenty one, based on the opinion of an observer regarding the hindlimb movements, joint movements, forelimb and hindlimb coordination, stepping, trunk position and stability, paw placement and tail position. The scale score is divided into three stages: (i) Early stage (score of 0–7): composed of isolated joint movements with little or no hindlimb movement; (ii) Intermediate stage (score of 8–13): intervals of uncoordinated stepping, and (iii) Late stage (score of 14–21): forelimb and hindlimb coordination.

Since the BBB score is determined by a person based on his own experience and expertise, the major limitation of the this and other qualitative methodologies is the reduced reproducibility and non-satisfactory sensitivity for some cases of locomotion studies [13].

To overcome this challenge, an automated gait analysis method based on the analysis of paw-floor contact was developed by Hammers et al. [14], which allows the quantification of a number of locomotion variables regarding the step cycle, pressure applied, and stance phases.

Another approach consists of performing an analysis of the patterns generated by the configuration of the rat’s extremity joints during their movement, either using a forced or an unforced methodology (e.g., [15–18]. These approaches rely on the use of video sequence recording at high speed (i.e., in general above 90 frames per second) that are analyzed through the inspection of the angles between the joints of the extremity of interest obtained by manual annotations of the extremity along the recorded frames. The curves corresponding to changes of the joint angles with respect to time can then be used to compare the gait patterns of different experimental rat groups [7].

However, the great challenge of this approach is the significant amount of effort required for the manual annotation of the frames of the videos. The person who does the annotations may also perform poorly due to lack of experience or fatigue.

Therefore, it is necessary to the automate the annotation process and the subsequent computation of angle variations with respect to time. In this work, we present a method that is based on the use of a bio-inspired algorithm that fits a kinematic model of the hind limb of rats to a binary image corresponding to the segmented marker of an image of the rat while walking. The bio-inspired algorithm combines a genetic algorithm for a group of the optimization variables with a local search for a second group of the optimization variables. Our results indicate the feasibility of employing the proposed approach for the automatic annotation and analysis of the locomotion patterns of the posterior extremities of laboratory rats.

## Methods

**Fig. 1:**
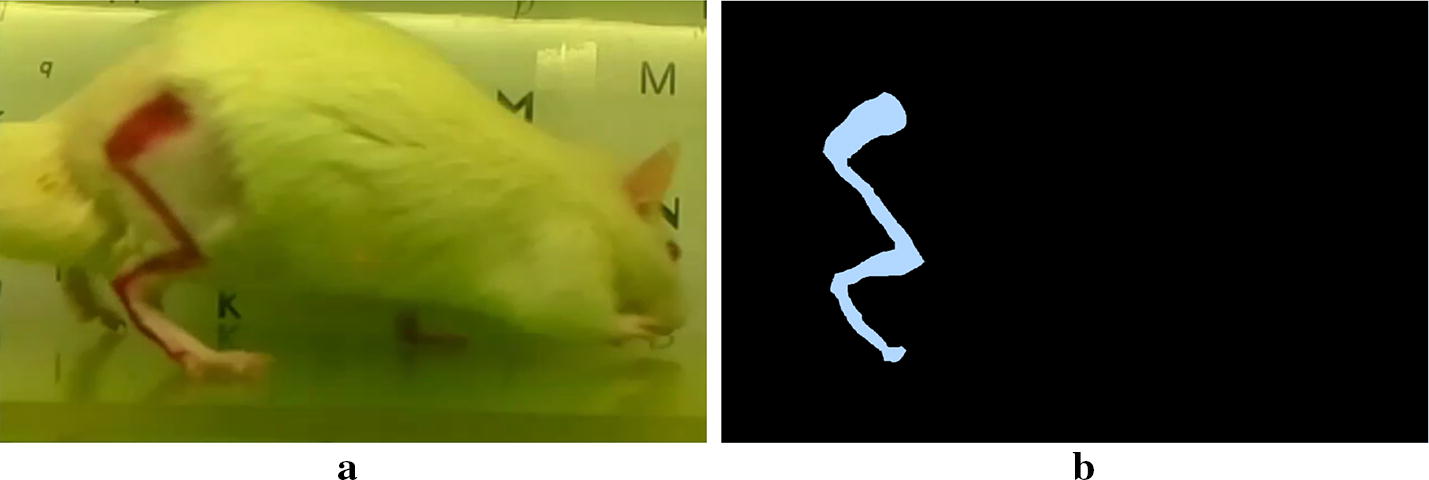
**a** Example of marks placed on the skin of the hind limb of a laboratory rat, and **b** the binary image corresponding to the marks

**Fig. 2:**
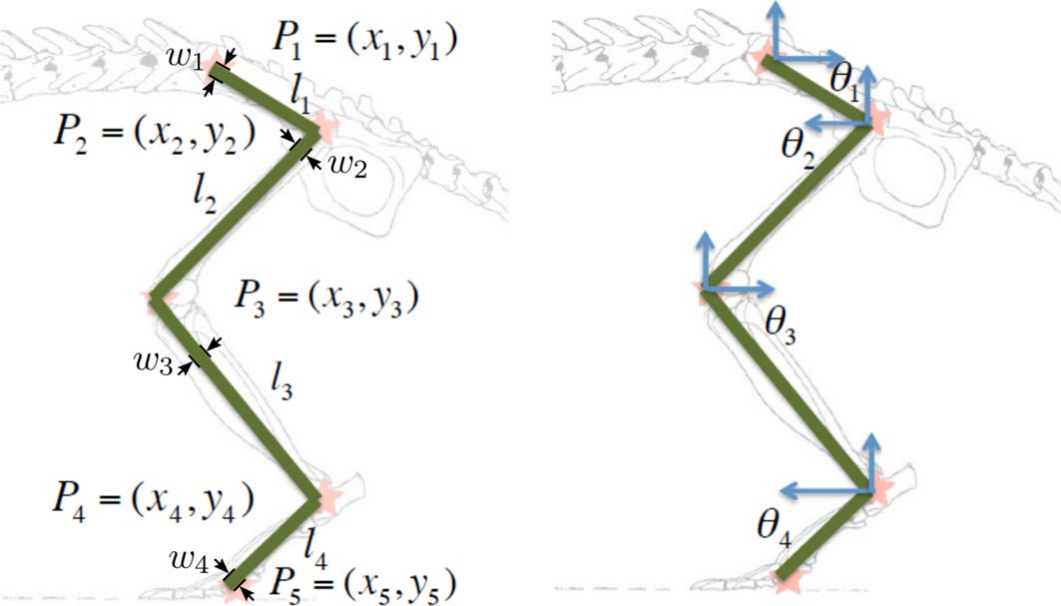
Kinematic model of the rat hind limb

The proposed method is based on the adjustment of a rat hind limb kinematic model to a frame in a rat’s gait video sequence, where the hind limb of the rat has been marked with line-marks corresponding to the the rat’s leg bones ( Fig. 1a). The model consists of points representing the joints named as $P_1,\ldots,P_5$, the lengths of each element of the leg $l_1,\ldots,l_4$, and the angle of each element by $\theta _1,\ldots,\theta _4$. Although leg widths are meaningless for the kinematic model, there are useful for the sake of fitting the virtual model to the video frames. Thus, they are marked with $w_i$ in Fig. 2.

Given the upper-most point $P_1=(x_1,y_1)$, the rest of the points can be obtained by:

1 $$\begin{aligned} x_{n} = x_{n-1} + l_{n-1}(cos(\theta _{n-1})) \end{aligned}$$

2 $$\begin{aligned} y_{n} = y_{n-1} + l_{n-1}(sin(\theta _{n-1})) \end{aligned}$$

for $$n=2...5$$.

Thus, the hind limb model for a given position can be represented as a fourteen-dimensional vector:

3 $$\begin{aligned} X=[x_1,y_1,l_1,l_2,l_3,l_4,\theta _1,\theta _2,\theta _3,\theta _4, w_1, w_2,w_3,w_4] \end{aligned}$$

where $l_i$, with $i \in \{1..4\}$, are the lengths of the leg elements, and $\theta _i$ and $w_i$ are angles and widths of the elements, respectively (Fig. 2).

Therefore, the problem consist of finding a vector of parameters $X^*$ which produces a binary image *E*(*x*, *y*) which fits the most (i.e., is the most similar) to a binary image *S*(*x*, *y*) representing the hind limb marks on a given frame (Fig. 1b). A fitting score *f*(*X*) can be obtained by:

4 $$\begin{aligned} f(X)=\frac{TP+TN}{P+N}. \end{aligned}$$

Where *TP* is the number of *true positives*, which are the number of white pixels in *E*(*x*, *y*) that are white in *S*(*x*, *y*). *TN* is the number of *true negatives*, negatives, which are the number of black pixels in *E*(*x*, *y*) that are black in *S*(*x*, *y*). *P* and *N* are the total number of *positives* and *negatives* in *S*(*x*, *y*). This measure is known, in the context of binary classifiers, as *accuracy*. The maximum value of the objective function is 1.

In this work, we propose to compute the solution $X^*$ using a two-step method that consists of a genetic algorithm for finding the values of the initial point $(x_1,y_1)$ and lengths and a local search algorithm for the other variables.

### Genetic algorithm

We implemented the Elitist Non-Dominated Sorting Genetic Algorithm II (NSGA-II) [19], since it has demonstrated outstanding performance and is currently one of the most widely used evolutionary algorithms. The initial population $Z^{z=0}$ is generated using aleatory values for the uppermost point $P_1$ and lengths $l_i$ of the kinematic model. $P_1$ is selected from the uppermost white pixels. Therefore, we use a single optimization variable (the index of the pixel), A local search is then performed to find the best angles $(\theta _i)$ and widths $(w_i)$ for the given uppermost point and section lengths. Each individual of the population $X^{z}_{c}$ is evaluated on the objective function and the result is established as the fitness score *F*. Notice that the evaluation procedure is, actually, the local search procedure. The genetic algorithm provides the initial point and length, while the local search find angles and widths. In contrast with most hybrid algorithms, our global and local procedures affect different optimization variables. After evaluating the population, the best individual $X^{*z}$ is selected to be incorporated into the next generation population $Z^{z+1}$ (elitism). The rest of the individuals are selected for reproduction using binary tournament [20], which consists of several comparisons of objective function value (“tournaments”) among a set of individuals chosen at random from the population. The winner of each tournament (the one with the best fitness) is selected for reproduction.

The reproduction of two selected individuals $X^{z}_{c_1}$ and $X^{z}_{c_2}$ is performed using the simulated binary cross-over(SBX) method [19], which consists of the generation of a random number *u* in the range of [0, 1] that is is used for determining the contribution $\bar{\beta }$ of the characteristics from each parent to each children as:

5 $$\begin{aligned} \bar{\beta } = {\left\{ \begin{array}{ll} (2 \cdot u)^{\frac{1}{n_{c}+1}} &{} \quad \text {if } u < 0.5\\ \left(\frac{1}{2(1-u)}\right)^{\frac{1}{n_{c}+1}} &{} \quad \text {otherwise}\\ \end{array}\right. } \end{aligned}$$

where $n_{c}=1$. A pair of children $X^{z+1}_{c_1}$ and $X^{z+1}_{c_2}$ are then generated according to the following equations:

6 $$\begin{aligned} X^{z+1}_{c_1}= & {} \frac{1}{2}\left[(X^{z}_{c_1} + X^{z}_{c_2}) - \overline{\beta }(X^{z}_{c_2}-X^{z}_{c_1})\right] \nonumber \\ X^{z+1}_{c_2}= & {} \frac{1}{2}\left[(X^{z}_{c_1} + X^{z}_{c_2}) + \overline{\beta }(X^{z}_{c_2}-X^{z}_{c_1})\right] \end{aligned}$$

Finally, to prevent the population from getting stuck in local minimums and maintain diversity in the exploration of the solution space, we employed the polynomial mutation method [21] on which the value of an element of an individual may change with a probability *m*. The new value is determined by a polynomial probability distribution: $P(\delta ) = 0.5(\eta _{m}+1)(1-|\delta |)^{\eta _{m}}$; $x_{i}^{U}$ and $x_{i}^{L}$ are the lower and upper bound of $x_i$ respectively. The mutated children together with the elite individual (best-known solution) become the new population. The loop of evaluation, elitism, selection, reproduction and mutation are repeated until a stopping criterion is met. In this work, the stopping criterion depends on the consecutive number of generations $N_{maxelite}$ in which the elite individual is not improved.

Algorithm 1 list the steps of the first part of the proposed approach based on the genetic algorithm.

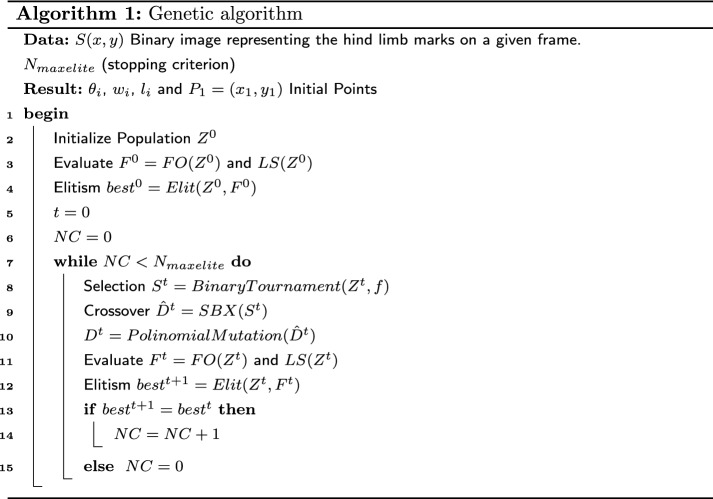



### Local search

The local search method intends to find the optimal values of $\theta _i$ and $w_i$ for each hind limb segment by searching in a given range of angular values $\theta ^{inf}_i$ and $\theta ^{sup}_i$, using the objective function in Eq. 4. The method consists of two stages of refinement, where in each of these, the search is performed with a higher precision in a reduced range.

The first step starts with an initial width of $w_0=10$. The method performs a search every four degrees of the angle in which the overlap of the line drawn in the image $D^{I}_{i}$ and the image *S*(*x*, *y*) is maximized, according to Eq. 4. The best angle found is stored in the variable $\theta ^{I}$.

A second search is performed around this last value, every one degree. For each angle in the second search, we intend to improve the width of the segment looking for a new width. The best angle found is stored in the variable $\theta ^{I}$, and the best width in the variable $w_{n-1}^{II}$. Finally, the values of the width and the angle are stored and the limits updated. The procedure is repeated until all angles and widths are estimated. Algorithm 2 lists the steps of the proposed local search method.

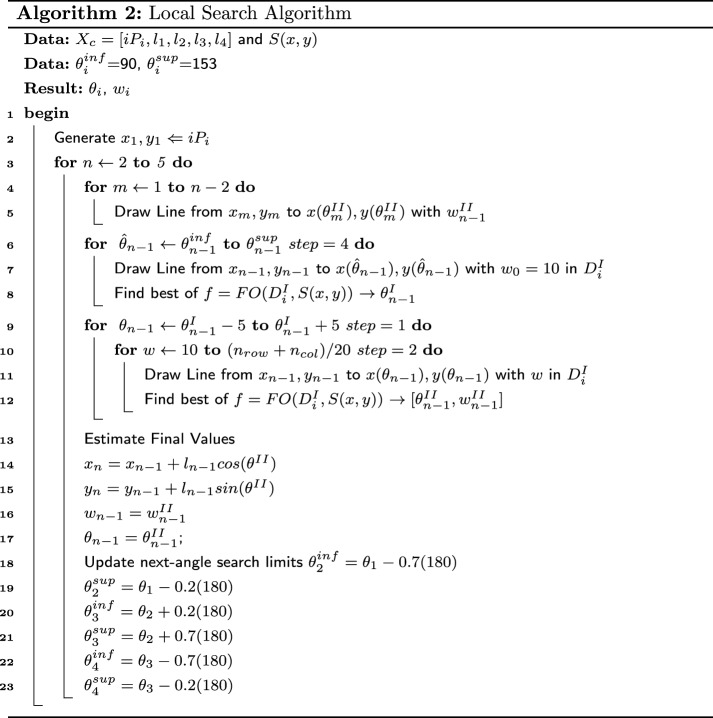



### Leg pendulum-like movement computation

A possible way to quantitatively evaluate the gait properties of rats is by comparing the curves that are generated by the movement of the extremities during the steps. One of the curves that can be employed to perform a comparison of hind limbs locomotion is the leg pendulum-like movement (PLM) of the hind limb which corresponds to the angle between the the 5th lumbar vertebra and fifth metatarsophalangeal joint (first and last points) [7]. In this work, we computed this angle for each frame, interpolated missing angles in case of non convergence of the algorithm, and then applied a robust weighted moving average filter with an outliers parameter of $6\sigma$.

## Results

Six hundred frames corresponding to steps from fourteen laboratory rats marked on their hind limb using a red water-based non-toxic marker were obtained from video sequences recorded at 90 frames per second at a resolution of 640 x 480 pixels. Segmentation of the marked region during the steps were obtained automatically by converting each frame into an HSV color space and then applying a threshold over the Hue channel of the image.

**Fig. 3:**
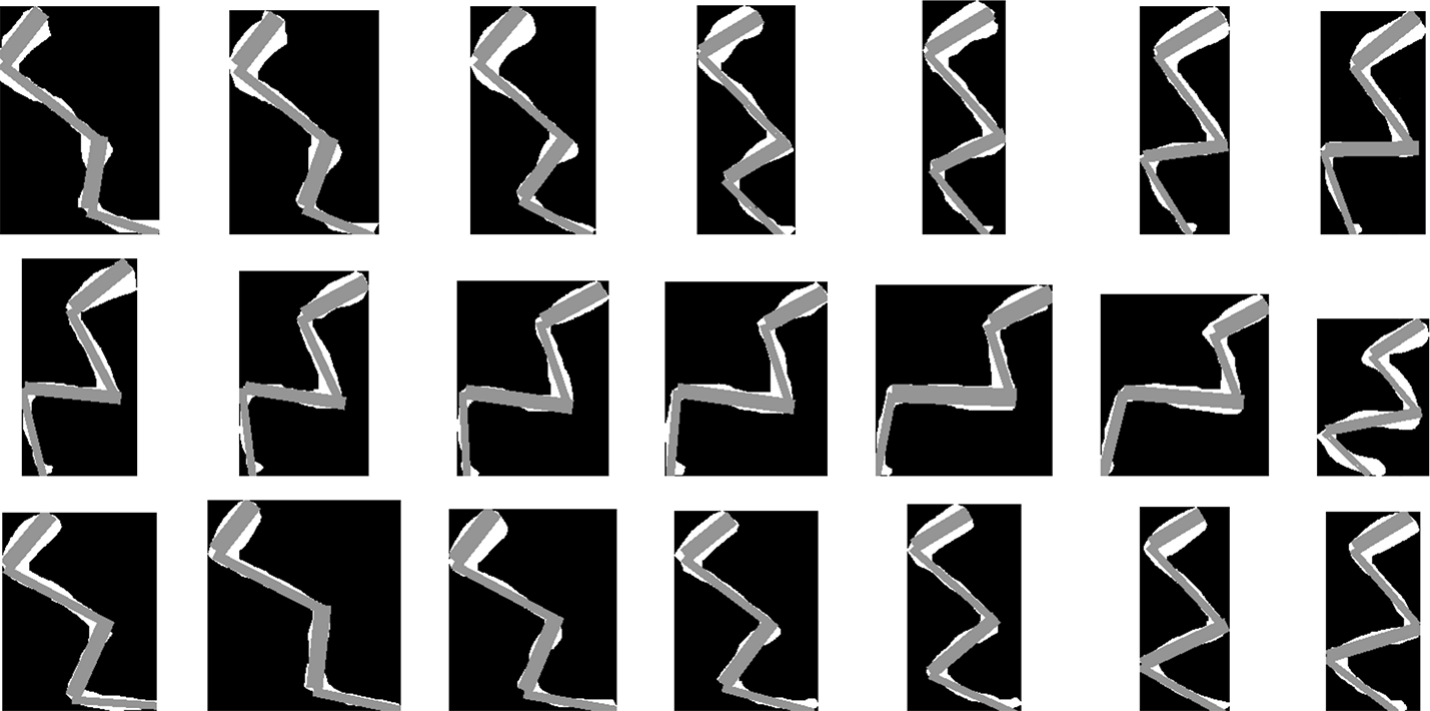
Examples of the kinematic model adjustments over the binary images corresponding to the hind limb marks of a rat employing the proposed method

The genetic algorithm with local search is applied on each frame using a population size of 400, mutation probability of 0.11, crossover probability of 0.9, number of elite individuals preserver through a generation is 3, and value of $N_{maxelite}=8$ as stopping criterion. Figure 3 depicts an example of the best adjusted model on 21 frames corresponding to a step of a rat. Note that the kinematic model adjusted by the proposed method is very similar to the segmented marked region.

**Fig. 4:**
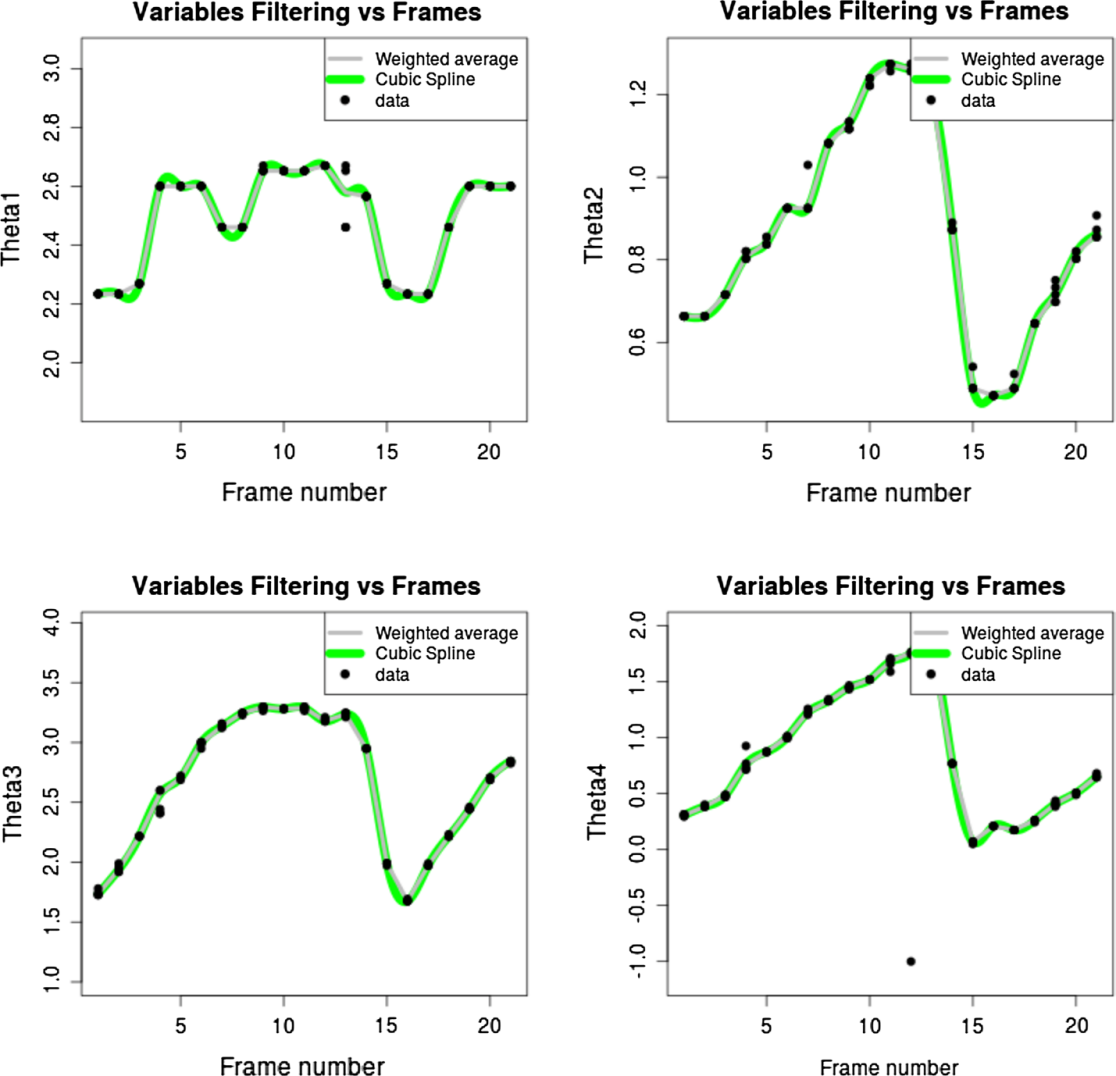
From up to down, left to right angles. Dots are $\theta _1, \theta _2, \theta _3$ and $\theta _4$ estimated values from five executions per frame. The gray line is a weighted mean, each value is weighted by the normalized objective function (the normalized objective function values from the five executions sums to one) . The green line is a cubic-spline interpolation of the weighted mean

Table 1 depicts the statistics of the variation of the results of five independent executions of the proposed method on 100 randomly selected frames using Bootstrapping and a re-sampling parameter of $N=1000$. Figure 4 depicts an example of the computed angles for the four angles in five independent executions (dots), along with a weighted mean (gray line), and a cubic spline interpolation (green line). Note that the computed angles have a small dispersion, which indicates that the algorithm is robust with respect to the variability of the rat’s hind limbs.

**Fig. 5:**
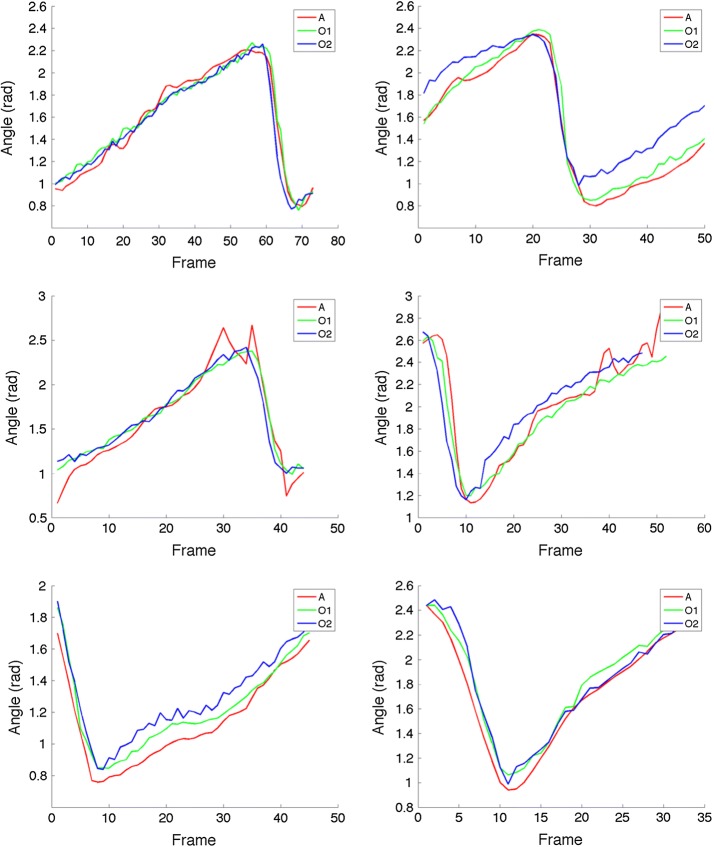
Examples of the the leg pendulum-like movement of the hind limb obtained with the proposed method (A), and with the annotations of two observers (O1 and O2)

The feasibility of the proposed method to be used to perform the quantitative analysis is evaluated by comparing the PLM of the hind limb of each rat’s step obtained with the proposed method (A), with the corresponding curve obtained from the annotations of two observers (O1 and O2) on the image sequences (Fig. 5). Note that, in general the three curves are very similar in the pattern they describe, with exception of some localized differences.

**Table 1:** Statistics of the variation of the results of the adjusted kinematic model employing the proposed method five times on 100 randomly selected frames using Bootstrapping and a re-sampling parameter of $N=1000$

Variable	Mean difference (std)	95% Confidence interval
$l_1$ (pixels)	2.39 (4.87)	1.95–3.02
$l_2$ (pixels)	1.86 (3.56)	1.56–2.35
$l_3$ (pixels)	3.32 (6.27)	2.65–4.12
$l_4$ (pixels)	12.59 (10.48)	11.41–13.79
$\theta _1$ (rad)	0.02 (0.06)	0.018–0.034
$\theta _2$ (rad)	0.01 (0.03)	0.013–0.022
$\theta _3$ (rad)	0.01 (0.02)	0.013–0.019
$\theta _4$ (rad)	0.36 (1.58)	0.210–0.582

The similarity of the PLM curves for each rat step obtained with the proposed method and with the observer’s annotations was assessed by the computation of the Frechet distance [22], which is a measure of similarity between curves that takes into account the location and ordering of the points along the curves. The mean difference between A and O1 was 0.453 ± 0.147, the difference between A and O2 was 0.544 ± 0.25, while the difference between the O1 vs O2 was 0.298 ± 0.093. We performed a two-sample T-test with a significance level of 0.05 to determine if the differences in the performance between the automatic method and the human observers was comparable. The results for the comparison of A vs O1 and O1 vs O2, the null-hypothesis was rejected with a value of $p = 0.068$, which indicate that there are not statistically significant differences between the performance of the proposed method and Observer one. However, for the case of the comparison comparison of A vs O2 and O1 vs O2, there was not enough statistical evidence to reject the null-hypothesis ($p = 0.00524$) which suggests that the differences of the proposed method with respect to the annotations of O2 are greater than the differences of O1 and O2.

## Discussion

The obtained results indicate the feasibility of employing the proposed method for the adjustment of the hind limb kinematic model to markers of the video frames corresponding to rat’s gait sequences. The obtained joint configuration could then be used to analyze the motion patterns during the steps, which, in turn, can be useful for performing quantitative evaluations of the effect of lesions and treatments on rats models.

In our experiments, we noted that the lengths and widths of the hind limb segments vary with respect to the step cycle instant. For instance, for all executions on frames in Fig. 3, the length’s means and standard deviations are $\mu _l=\{67.2, 108.09,94.35, 101.56\}$ and $\sigma _l=\{5.69, 5.493, 16.00, 16.82\}$ for {$l_1,l_2,l_3,l_4$} respectively, and the width’s means and standard deviations are $\mu _w=\{15.71, 15.56,15.35, 14.57\}$ and $\sigma _w=\{1.67, 1.62, 1.67, 2.48\}$ for {$w_1,w_2,w_3,w_4$}, respectively. All measurements are in pixels. This variation could be explained by the three dimensional movement of the leg which occurs in the direction of the cameras. We believe that it could be possible to estimate the characteristics of this motion by comparing the obtained lengths and widths with the real proportions of these features. Another option could be to employ more than one camera at different known positions, then perform the model fitting, and finally estimate the 3D motion by triangulation of the known corresponding points.

Future work includes the addition of a kinematic model for the forelimbs, and the comparison of the results obtained with the proposed method with manual annotations performed by an observer over the same video sequences.

## Conclusion

We have presented a method that is based on the use of a bio-inspired algorithm that fits a kinematic model of the hind limb of rats to binary images corresponding to the segmented marker of images corresponding to rats’ gait. The obtained results indicate the feasibility of employing the proposed approach for the automatic annotation and analysis of the locomotion patterns of the posterior extremities of laboratory rats.
